# Pseudohyperkalemia: Hyperkalemia Cocktail or Alternative Diagnosis

**DOI:** 10.1155/2018/9060892

**Published:** 2018-07-24

**Authors:** Sanjay Prakash Neupane, Pratibha Sharma, Mahesh Mani Dangal

**Affiliations:** ^1^Internal Medicine, PeaceHealth Southwest Medical Center, 400 NE Mother Joseph Place, Vancouver, WA 98664, USA; ^2^Maimonides Medical Center, 4802 10th Avenue, Brooklyn, NY 11219, USA

## Abstract

**Introduction:**

Hyperkalemia is a commonly encountered clinical problem. Pseudohyperkalemia is believed to be an in vitro phenomenon that does not reflect in vivo serum potassium and therefore should not be treated. Here, we present a case who unfortunately underwent unnecessary treatment because of failure to detect the common lab abnormality of pseudohyperkalemia.

**Case Presentation:**

A 91-year-old female with a history of chronic lymphocytic leukemia presented to the emergency with nausea and vomiting 24 hours after her first chemotherapy with chlorambucil. Physical examination was overall unremarkable. She had a leukocytosis of 210 × 10^3^/*µ*L with 96% lymphocytes along with chronic anemia with hemoglobin of 8.1 g/dL. Her initial sodium and potassium levels were normal. During the clinical course, her potassium progressively worsened and failed to improve despite standard medical treatment. Patient ultimately underwent dialysis.

**Conclusions:**

Differentiating true hyperkalemia from pseudohyperkalemia is very important in selected group of patients to avoid unnecessary medications, higher level of care, and unnecessary procedure including dialysis. We want to emphasize the importance of simple yet profound knowledge of technique of blood draws and basic metabolic panel processing for every clinician in day-to-day practice.

## 1. Introduction

Hyperkalemia is a commonly encountered clinical problem. It can be fatal if not treated emergently. Common cause of hyperkalemia is due to decreased urinary potassium excretion in the setting of acute and chronic kidney injury. Less common cause includes increased potassium release from cells including pseudohyperkalemia, increased tissue catabolism, and insulin deficiency. We present here a 91-year-old female with a history of chronic lymphocytic leukemia (CLL) presenting with elevated serum potassium level that unfortunately underwent unnecessary treatment because of failure to detect the common lab abnormality of pseudohyperkalemia.

## 2. Case Presentation

A 91-year-old Caucasian female with a history of CLL diagnosed 14 years ago, hypothyroidism, glaucoma, and severe osteoarthritis of spine presented to ER, 24 hrs after receiving first chemotherapy for her CLL with chlorambucil in the setting of recent worsening of lymphocytosis, anemia, and exertional dyspnea, with complaints of nausea and vomiting after chemotherapy. There was no diarrhea, abdomen pain, fever, and chills. She had completed a course of Bactrim for UTI a week ago. There were no recent changes in home medications. Vital signs were within normal limits, and systemic physical examination was unremarkable except for dry mucous membrane in ER. She received a liter of normal saline bolus and was started on maintenance normal saline at 75 ml/hr. She started to feel short of breath and wheezy while in ER. Her oxygen saturation dropped to 85% on room air, which was treated with bronchodilators. Upon initial workup, she was found to have leukocytosis of 210 × 10^3^ cells/*μ*L with 96% lymphocytes, along with chronic anemia with hemoglobin of 8.1 and hematocrit of 28. Her electrolytes and renal function were normal with Na of 137 meq/L, K of 4.6 meq/L, BUN of 15 mg/dL, and Cr of 0.8 mg/dL. CXR was unremarkable. Liver function tests were within normal limits. Uric acid was 2.8 mg/dL, and phosphorus was 2.9 mg/dL. The patient got progressively short of breath overnight after admission and was hypoxic. She was, therefore, evaluated for pulmonary embolism based on her risk profile. CTA chest was negative for pulmonary embolism. This revealed diffuse centrilobular emphysematous changes and bibasilar atelectasis. Patient's care was escalated to intensive care, and noninvasive ventilation was initiated. Repeat basic metabolic panel this time revealed K of 6.6 meq/L. Rest of labs including creatinine were essentially within normal limits. The basic metabolic panel was repeated again in 3 hrs, which revealed K of 8.5. EKG did not demonstrate peaked T waves, prolonged QRS interval, or evidence of heart block. She was treated with intravenous calcium gluconate, albuterol nebulization, IV insulin, IV dextrose, IV Lasix, and sodium polystyrene. Her K remained persistently high in 8 meq/dl most of the day despite medical management. When her K rose to 9.1 overnight, a decision was made to proceed to emergent dialysis. Interestingly, her creatinine remained stable throughout, and she was not oliguric. She received 4 hrs of hemodialysis with 2 K bath. Immediately after dialysis, she had a run of supraventricular tachycardia with a heart rate of 130, which did not improve with adenosine. Her potassium by arterial blood gas was 3.0 meq/dL, and K on basic metabolic panel was 3.1 an hour after hemodialysis. Potassium was supplemented intravenously, and she was also loaded with digoxin following which she converted to sinus rhythm. She sustained a demand ischemia with troponins going up to 10 after this event. No cardiac catheterization or ischemic workup was done, as she was asymptomatic after resolution of SVT. Her plasma K was between 3.5 and 4.7 for next six days prior to discharge home from hospital.

## 3. Discussion

Pseudohyperkalemia is believed to be an in vitro phenomenon that does not reflect in vivo serum potassium and therefore should not be treated. Laboratory finding of pseudohyperkalemia was first described in 1975 in two CLL patients with WBC counts above 600 k/*µ*L [1]. Differentiating true hyperkalemia from pseudohyperkalemia is very important in selected group of patients to avoid unnecessary medications, higher level of care like admitting in ICU, and unnecessary procedures including dialysis. In patients with increased cell counts, RBC, WBC, or platelets, there are several factors that play a role in mechanical disruption of blood cells [1–3]. Before the start of heparinization of the collected blood specimen, this was believed to be due to the clotting process inducing in vitro release of potassium from leukocytes. Potassium is now measured in plasma or in heparinized tubes, clotting is unlikely to be causal, but lysis of cells still can occur [1]. Use of vacuum tubes, pneumatic tube transportation, prolonged incubation, tourniquet use, and processing of specimens through centrifugation have all been implicated as causing lysis of cells and releasing serum potassium [4–6]. In patients with CLL, the leakage of potassium from elevated fragile white blood cells results in falsely elevated serum potassium. Lack of metabolic fuels leading to impaired sodium/potassium adenosine triphosphatase activity may contribute to release of potassium from a large number of white cells [4, 7]. In our case, all the basic metabolic panel testing was done with the standard technique on plasma. After realization of a spurious phenomenon, on subsequent blood draws, mechanical factors were minimized by avoiding tourniquet, decreasing specimen transport delay, and rapidly processing the specimen. ABG was also performed on one occasion. Arterial blood draws might be more accurate than venous blood draws in the similar patients simply due to the fact that arterial samples are less susceptible to stressors because of quick processing and the lack of use of tourniquet. In our case, hemodialysis was felt as the appropriate intervention because of the refractory hyperkalemia. Unfortunately, failure to recognize the possibility of pseudohyperkalemia resulted in intervention that could have led to significant morbidity and mortality in this 91-year-old.

## 4. Conclusions

Pseudohyperkalemia is not an uncommon entity. However, at many times, we as clinicians tend to go behind numbers rather than seeing the whole picture of the patient. There were several clinical clues pointing towards alternate explanation for the abnormal lab value. The fact that our patient's creatinine being persistently normal, the patient continued to make urine and no EKG changes despite high potassium level should have prompted to think outside of true hyperkalemia. As a clinician, we need to be very attentive and consider several physical and technical factors before interpreting any abnormal lab value. Through this case report, we want to emphasize further on the importance of knowledge of this simple concept related to techniques of blood draws, basic metabolic panel processing, and correlating the lab value with clinical presentation on every clinician in day-to-day practice [8, 9].

## Data Availability

All data generated or analyzed during this study are included in this article.

## Abbreviations

CLL: Chronic lymphocytic leukemia
ER: Emergency
UTI: Urinary tract infection
Na: Sodium
K: Potassium
BUN: Blood urea nitrogen
Cr: Creatinine
CXR: Chest X-ray
CTA: CT angiogram
EKG: Electrocardiogram
IV: Intravenous
SVT: Supraventricular tachycardia
WBC: White blood cells
RBC: Red blood cells
ABG: Arterial blood gas.

## References

[1] Ruddy K. J., Wu D., Brown J. R. (2008). Pseudohyperkalemia in chronic lymphocytic leukemia. *Journal of Clinical Oncology*.

[2] Colussi G., Cipriani D. (1995). Pseudohyperkalemia in extreme leukocytosis. *American Journal of Nephrology*.

[3] Nijsten M. W., de Smet B. J., Dofferjoff A. S. (1991). Pseudohyperkalemia and platelet counts. *New England Journal of Medicine*.

[4] Chawla N. R., Shapiro J., Sham R. L. (2009). Pneumatic tube “pseudo tumor lysis syndrome” in chronic lymphocytic leukemia. *American Journal of Hematology*.

[5] Rifkin S. I. (2011). Pseudohyperkalemia in patients with chronic lymphocytic leukemia. *International Journal of Nephrology*.

[6] Kellerman P. S., Thornbery J. M. (2005). Pseudohyperkalmia due to pneumatic tube transport in Leukemic patient. *American Journal of Kidney Diseases*.

[7] Colussi G. (2006). Pseudohyperkalemia in leukemias. *American Journal of Kidney Diseases*.

[8] Don B. R., Sebastian A., Cheitlin M., Christiansen M., Schambelan M. (1990). Pseudohyperkalemia caused by fist clinching during phlebotomy. *New England Journal of Medicine*.

[9] Asirvatham J. R., Moses V., Bjornson L. (2013). Errors in potassium measurement: a laboratory perspective for the clinician. *North American Journal of Medical Sciences*.

